# Paroxysmal Atrial Fibrillation during Spasm Provocation Test with Acetylcholine: Clinical Characteristics of Patients and Effect on Coronary Microvascular Function Measurements

**DOI:** 10.31083/RCM26456

**Published:** 2025-02-12

**Authors:** Hiroki Teragawa, Chikage Oshita, Yu Hashimoto, Shuichi Nomura

**Affiliations:** ^1^Department of Cardiovascular Medicine, JR Hiroshima Hospital, 732-0057 Hiroshima, Japan

**Keywords:** acetylcholine, coronary flow reserve, coronary spasm, index of microcirculatory resistance, paroxysmal atrial fibrillation, spasm provocation test

## Abstract

**Background:**

Atrial fibrillation (AF) is a complication that occurs following a spasm provocation test (SPT) with acetylcholine (ACh). However, the characteristics of patients with AF remain unclear. Furthermore, the association of AF with the outcome of the coronary microvascular function test (CMFT) is unknown. This study aimed to evaluate whether patients with angina with non-obstructive coronary artery disease (ANOCA) who developed AF during SPT with ACh had any clinical characteristics. Additionally, we assessed the association of AF with the CMFT results.

**Methods:**

We included 123 patients with ANOCA who underwent SPT and CMFT. We defined AF as AF during ACh provocation. The coronary arteries that demonstrated AF before CMFT were defined as AF vessels (n = 21) and those in sinus rhythm (SR) were defined as SR-1 vessels (n = 165). Vessels that were restored to sinus rhythm immediately following AF were defined as AF-SR vessels (n = 29) and those that remained in sinus rhythm for some time were defined as SR-2 vessels (n = 136). Coronary flow reserve (CFR) and index of microcirculatory resistance (IMR) were obtained, and CFR of <2.0 and/or IMR of ≥25 were diagnosed as coronary microvascular dysfunction (CMD).

**Results:**

Of the 123 patients, 31 (25%) had AF but with no characteristic patient background. CFR was significantly lower in AF vessels than in SR-1 vessels (*p* = 0.035) and IMR did not differ between the two groups (*p* = 0.918). A study of the three groups that included AF-SR vessels revealed that IMR tended to be lower in AF-SR vessels than in the SR-2 and AF vessels (*p* = 0.089), and that the frequency of IMR of ≥25 was significantly lower than in the other two groups (*p* = 0.016).

**Conclusions:**

AF occurred in 25% of SPTs with ACh, but the predictive clinical context remains unclear. Our results indicated that AF may affect the outcome of the CMFT. Thus, decisions for CMD management should be made with caution in the presence of AF.

## 1. Introduction

Angina with non-obstructive coronary artery disease (ANOCA) is a prevalent condition that has recently received increased attention [1, 2]. The leading causes of ANOCA are vasospastic angina (VSA), coronary microvascular dysfunction (CMD), or both [1, 2]. The coexistence of both underlying causes has a poor prognosis [3]. Treatment is superior for improving subjective symptoms in ANOCA with a known cause treated with pharmacological therapy [4]. Consequently, VSA is recommended to be identified using spasm provocation testing (SPT) and CMD with the coronary microvascular function test (CMFT).

SPT and CMFT are widely recognized for testing [1, 2]. However, which test should be performed first remains unestablished and also varies based on factors such as subjective symptoms, institutional experience, and policies. CMFT requires maximally dilated coronary arteries; thus, nitroglycerin (NTG) preadministration is mandatory. However, NTG preadministration may have a significant effect on SPT results [5]. Furthermore, patients with CMD (especially those with reduced coronary flow reserve [CFR]) demonstrate a poor prognosis [6], but VSA also causes cardiac arrest [7, 8]. Further, beta-blockers (the main treatment for CMD) [1] are restricted for treating VSA when administered alone [9]. Therefore, VSA must be diagnosed before beta-blocker administration. Hence, SPT is frequently administered before CMFT in Japan, including our institution.

Conversely, one of the complications of SPT with acetylcholine (ACh) is paroxysmal atrial fibrillation (PAF) [10, 11]. Clinical characteristics that cause PAF during SPT remain unclear. Furthermore, atrial fibrillation (AF) may affect CMFT testing, especially in the CFR [12, 13, 14, 15]. Furthermore, the effect of PAF on CMFT outcomes needs to be identified. We investigated the clinical characteristics of patients who underwent SPT and CMFT to investigate the etiology of ANOCA, the clinical characteristics of patients who developed PAF, and the association of PAF occurrence with CMFT outcomes.

## 2. Materials and Methods

### 2.1 Study Participants

**Fig. 1.**
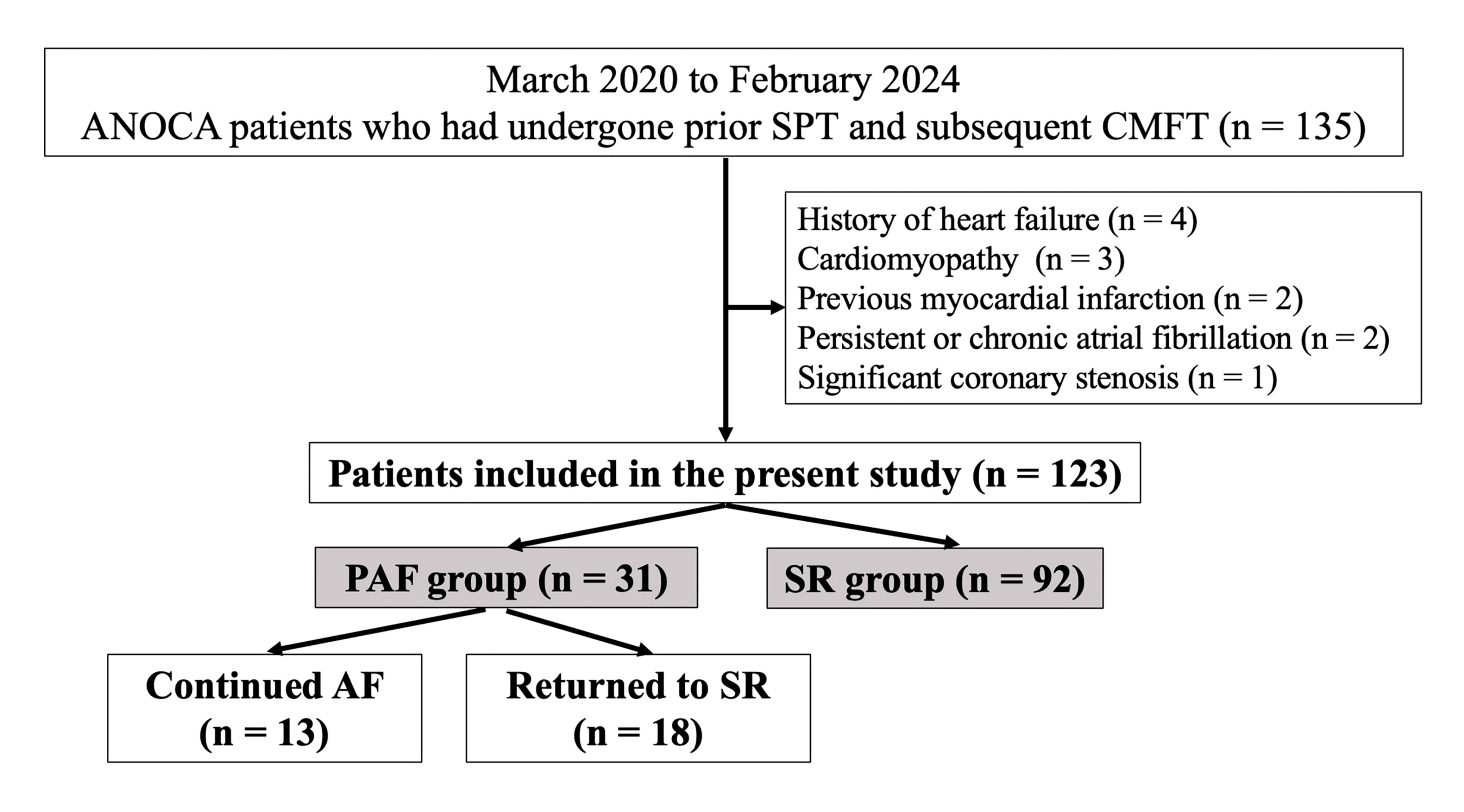
**Flowchart of the study protocol (per patient)**. Abbreviations: AF, atrial fibrillation; ANOCA, angina with non-obstructive coronary artery disease; CMFT, coronary microvascular function test; PAF, paroxysmal atrial fibrillation; SPT, spasm provocation test; SR, sinus rhythm.

This retrospective observational study involved 135 patients who underwent prior SPT and subsequent CMFT at our institution from March 2020 to February 2024 to evaluate the ANOCA endotype. Patients in whom at least one coronary artery vessel could be assessed by SPT and CMFT were included. Patients who underwent percutaneous coronary intervention (PCI) were included if they had any anginal pain without concurrent significant stenosis. This study excluded patients who had heart failure (n = 4), cardiomyopathy (n = 3), previous myocardial infarction (n = 2), and significant coronary stenosis (% stenosis >50%) as well as those with persistent or chronic AF at admission (n = 2). This study enrolled 123 patients (Fig. 1). All patients signed written informed consent for SPT and coronary angiography (CAG), and consent was confirmed with an opt-out method on the homepage (http://www.jrhh.sakura.ne.jp/annnai/torikumi.html) because of the retrospective study design. The Ethics Committee of our institution approved the study protocol (2024-16).

### 2.2 SPT and CMFT

The SPT procedures utilized at our institution have been previously published [16]. SPT was conducted after the conventional diagnostic CAG test and used a percutaneous brachial or radial route with a 5 French (Fr) sheath and a diagnostic Judkins-type catheter. A 5-Fr gauge temporary pacing catheter (Bipolar Balloon Catheter, B. Braun, Melsungen, Germany) was introduced into the right ventricle and set to 50 beats per minute through the internal jugular vein or medial cubital vein*. *The left coronary artery (LCA) was injected after the initial CAG, with 50, 100, and 200 µg of ACh for a duration of 20 s, with a 3-min gap between each injection. CAG was conducted either immediately after the maximum ACh administration or following the coronary spasm provocation. A dose of 0.3 mg of NTG was immediately delivered to the coronary artery if the introduction of ACh into the LCA induced prolonged contractions of the coronary arteries or caused unstable hemodynamics. Approximately 20, 50, and 80 µg of ACh were injected into the right coronary artery (RCA) for 20 s, with 3-min intervals between each dose, after inducing spasms in the LCA. Ergonovine maleate (EM, Fuji Pharma, Tokyo, Japan) was administered intra-coronarily in patients with negative responses to ACh provocation, as described in an earlier study [16]. The attending physician had the authority to decide on EM administration. CAG was repeated by injecting NTG into the coronary artery after conducting all the provocation tests for the LCA and RCA. The outcome was classified as “not diagnosed” (ND) if the subsequently conducted SPT produced a negative result after the NTG injection.

The methods used for the CMFT have been previously detailed [16]. A PressureWire X Cabled guidewire manufactured by Abbot Laboratories in Abbot Park, IL, USA, was used with a pressure-temperature sensor tip. An Abbott Vascular RadiAnalyzerTM Xpress (Santa Clara, CA, USA) was employed to evaluate the parameters. A PressureWire was attached to the distal portion of the left anterior descending coronary artery (LAD) and RCA, and three 3 mL of saline injections were administered at room temperature to establish a thermodilution curve for measuring the resting mean transit time (Tmn). Adenosine triphosphate (ATP) was intravenously administered through the peripheral veins at 160 µg/kg/min to stimulate blood flow (hyperemia). The proximal aortic pressure (Pa), distal pressure (Pd), and Tmn were measured during maximum hyperemia. The fractional flow reserve (FFR) was determined by calculating the lowest average of three consecutive beats under stable hyperemia. CFR was calculated as the ratio between the resting Tmn and the hyperemic Tmn. The index of microcirculatory resistance (IMR) was calculated during hyperemia with the formula Pd × Tmn. We adjusted the aortic pressure in the catheter and the pressure obtained using the PressureWire to minimize the effects of pressure drift before monitoring the measurements in each coronary artery. No disparity was revealed between the pressure recorded after removing the pressure wire and the aortic pressure.

### 2.3 Definition and Parameters Associated with the CAG, SPT, and CMFT

The methodology for confirming the diameter of the coronary artery has been previously described [16]. Segments demonstrating both spasticity and atherosclerosis were selected for the quantitative study. The study was conducted with the mean values derived from the three measurements. The percentage deviation from the baseline angiographic data was utilized to quantify the alterations in the coronary artery diameter in response to ACh and NTG infusions. Atherosclerotic lesions were categorized as those with stenosis of >20%. The study investigated the occurrence of myocardial bridging (MB), which was characterized as a decrease of >20% in coronary artery diameter during systole [17].

Coronary spasm was the epicardial coronary artery narrowing of >90%, as seen on angiography during SPT. Furthermore, the presence of recognizable chest discomfort and/or aberrant ST-segment deviation on electrocardiography (ECG) was suggestive of coronary spasm [2]. The American Heart Association defines focal spasm as the temporary coronary artery narrowing by >90%, which exclusively happens inside a single isolated coronary segment [18]. Diffuse spasm is a medical disease characterized by coronary artery narrowing in two adjacent segments and affects >90% of the arteries [18]. The exact time at which consecutive SPT values became negative after NTG administration to a single coronary artery could not be determined. The study categorized the ACh levels as low (L), moderate (M), and high (H), which corresponded to 50, 100, and 200 µg, respectively, for LCA and 20, 50, and 80 µg, respectively, for RCA. Microvascular spasm (MVS) is the term utilized to describe the lack of angiographic coronary spasms together with specific chest discomfort and ST-segment and T wave ECG abnormalities during SPT [2]. CMD was defined as an IMR of ≥25 units and/or a CFR of <2.0 [1].

**Fig. 2.**
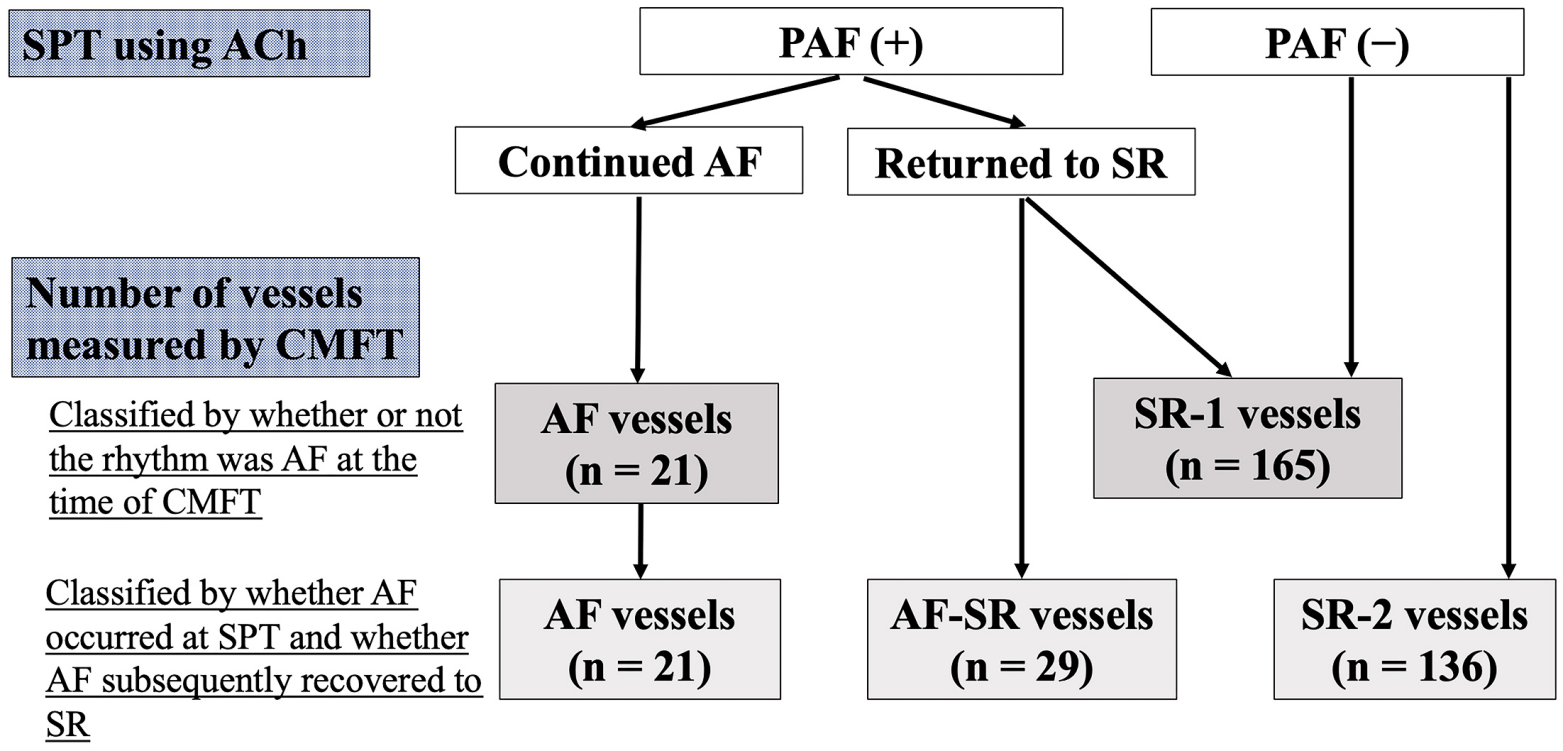
**Flowchart of the study protocol (per vessel)**. Abbreviations: AF, atrial fibrillation; CMFT, coronary microvascular function test; PAF, paroxysmal atrial fibrillation; SPT, spasm provocation test; SR, sinus rhythm; ACh, acetylcholine.

We strictly monitored AF occurrence during SPT. PAF is characterized as AF that was absent before SPT and emerged after ACh provocations, regardless of whether it persisted for >10 s, as verified by two observers. The patients were categorized based on the occurrence of PAF during SPT into the PAF and the sinus rhythm (SR) groups. Additionally, the dose of ACh administered and the vessels it was administered to were checked. We defined AF vessels as those in which CMFT was conducted when AF was sustained and SR-1 vessels as those in which SR was maintained if it was persistent during CMFT. AF-SR vessels are those with transient AF after ACh provocations but subsequently restored to SR, whereas those in SR throughout the investigation were SR-2 vessels (Fig. 2).

### 2.4 Parameters

Information on the familial history of coronary artery disease, present smoking behavior, and alcohol intake was collected [16]. The traditional definition of hypertension was utilized. The estimated glomerular filtration rate (mL/min/1.73 m^2^) was computed with an established formula [19]. Chronic kidney disease was evaluated based on the approved criteria. Both medication administration for dyslipidemia and having a low-density lipoprotein cholesterol level of ≥120 mg/dL were considered dyslipidemia indicators. Diabetes mellitus was defined as hemoglobin A1c values of ≥6.5%, a fasting blood sugar level of ≥126 mg/dL, and antidiabetic medication administration. Blood tests included C-reactive protein (mg/dL) and N Terminal-pro brain natriuretic peptide (NT-proBNP, pg/mL). Ultrasound cardiography was utilized to identify the left ventricular ejection fraction, the left ventricular mass index [20], and the left atrial diameter. The study assessed the ratio of the peak early diastolic velocity (E) to the peak early diastolic velocity at the septal side (e’) as an indicator of the left ventricular diastolic function [21]. The drugs administered upon admission were confirmed. We analyzed the amount consumed before termination although ceasing the administration of coronary vasodilators dilators 48 h before SPT is customary. The historical background of PCI was assessed.

### 2.5 Statistical Analyses

Continuous variables with normal distributions are expressed as means and standard deviations, and those variables with nonnormal distributions are presented as medians (interquartile ranges). Categorical variables are expressed as frequencies (%). Student’s unpaired *t*-test, the Wilcoxon signed-rank test, or Chi-square test were utilized to compare baseline characteristics between the groups, i.e., PAF and SR group or AF and SR vessels, or AF vessels, AF-SR vessels, and SR vessels. Logistic regression analysis was conducted to identify the factors that contributed to a CFR of <2.0. Spearman’s rank correlation coefficient was used for correlation analysis between the CFR, IMR, Tmn, and heart rate.

All statistical analyses were conducted with JMP (version 17; SAS Institute Inc., Cary, NC, USA). *p*-values of <0.05 denoted statistical significance.

## 3. Results

### 3.1 Patient Characteristics

**Fig. 3.**
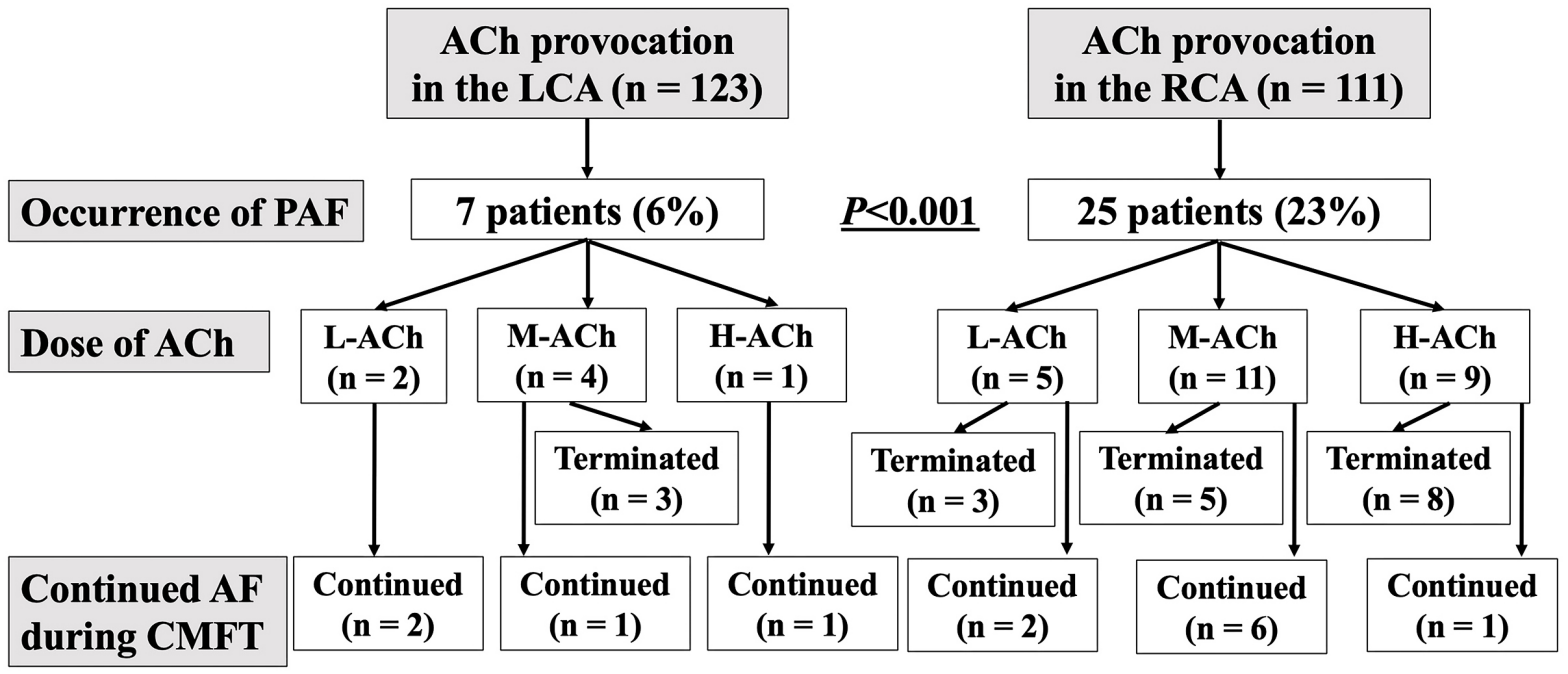
**Flowchart of PAF occurrence during SPT**. Abbreviations: SPT, spasm provocation test; ACh, acetylcholine; AF, atrial fibrillation; CMFT, coronary microvascular function test; H-ACh, high dose of acetylcholine; L-ACh, low dose of acetylcholine; LCA, left coronary artery; M-ACh, moderate dose of acetylcholine; PAF, paroxysmal atrial fibrillation; RCA, right coronary artery.

ACh provocation of LCA was performed in all 123 patients. ACh provocation was not performed in 12 patients due to the small size of the RCA or the judgment of the chief operator. ACh provocation of the RCA was performed in 111 patients. Additional use of EM provocation was performed in 26 (21%) patients. Hence, 31 (25%) patients had PAF (PAF group), consisting of 24 during RCA provocation, 6 during LCA provocation, and 1 in both coronary arteries (Fig. 3). One case in which PAF occurred in both LCA and RCA was calculated as LCA and RCA, respectively. PAF was reported in 7 (6%) of 123 patients with LCA provocation and 25 (23%) of 111 patients with RCA provocation, with PAF occurring more frequently during RCA provocation (*p* < 0.001). PAF occurrence was not related to ACh dosage. PAF was immediately discontinued in 18 (58%) of 31 patients, whereas AF persisted until CMFT in the remaining 13 (42%) patients. No difference in the cessation or persistence of AF was observed between LCA and RCA. AF was restored to SR on the same day of CAG after intravenous cibenzoline administration. No patient reported a PAF history.

No significant differences were found in the background of the three patient groups, including those with persistent AF until CMFT (n = 13), those who quickly recovered to SR (n = 18), and those who remained in SR (n = 92 patients) although not shown in the data.

Table 1 shows patient characteristics. No significant differences were found between the two groups. The frequency of female patients taking coronary vasodilators tended to be higher in the PAF group than in the SR group. Additionally, the E/e’ on ultrasound cardiography and NT-proBNP levels also tended to be lower than those in the SR group. Atherosclerotic lesion frequencies and PCI history were similar in the two groups, whereas the frequency of MB tended to be higher in the PAF group than in the SR group (*p* = 0.071). Positive SPT and CMD were comparable between the two groups.

**Table 1.** **Study participant characteristics**.

		SR Group	PAF Group	*p*-value
No. (%)		92 (75%)	31 (25%)	
Age (years)		65 ± 13	64 ± 14	0.736
Male/Female sex		43/49	9/22	0.084
Body mass index		24.5 ± 4.5	23.9 ± 4.1	0.513
Coronary risk factors (%)				
	Current smoker	12 (13)	2 (6)	0.378
	Hypertension	57 (62)	22 (71)	0.365
	Dyslipidemia	51 (55)	14 (45)	0.322
	Diabetes mellitus	14 (15)	4 (13)	0.753
Family history of CAD (%)		24 (26)	10 (32)	0.506
Alcohol drinker (%)		42 (46)	10 (32)	0.192
CKD (%)		27 (29)	7 (23)	0.137
Medications (%)				
	Coronary vasodilators	45 (49)	21 (68)	0.066
	RAS inhibitors	24 (26)	5 (16)	0.259
	Beta-blockers	8 (9)	5 (16)	0.244
	Statins	45 (49)	12 (39)	0.325
	Anti-platelet therapy	17 (18)	5 (16)	0.768
Blood chemistry parameters				
	C-reactive protein (mg/dL)	0.06 (0.03, 0.11)	0.04 (0.02, 0.07)	0.114
	eGFR (mL/min/1.73 m^2^)	67.5 ± 14.4	70.8 ± 13.6	0.268
	NT-proBNP (pg/mL)	71 (39, 142)	50 (33, 96)	0.062
Results of the UCG				
	LA diameter (mm)	34 ± 6	32 ± 5	0.125
	LVEF (%)	67 ± 7	68 ± 5	0.556
	LVMI (g/m^2^)	78 ± 16	80 ± 20	0.594
	E/e’	11.2 ± 4.8	9.6 ± 2.6	0.084
Results of the CAG, SPT and CMFT				
	Atherosclerosis (%)	43 (47)	15 (48)	0.834
	Myocardial bridging (%)	18 (20)	11 (35)	0.071
	History of PCI (%)	5 (5)	2 (6)	0.833
	Additional use of EM (%)	20 (21)	6 (19)	0.779
	Positive SPT (%)	65 (71)	20 (65)	0.523
	Presence of CMD (%)	62 (67)	18 (58)	0.346

Categorical variables are expressed as frequencies (percentages), and continuous variables are expressed as means ± standard deviations or medians (interquartile ranges). Abbreviations: CAD, coronary artery disease; CAG, coronary angiography; CKD, chronic kidney disease; CMD, coronary microvascular dysfunction; CMFT, coronary microvascular function test; eGFR, estimated glomerular filtration rate; EM, ergonovine maleate; LA, left atrial; LVEF, left ventricular ejection fraction; LVMI, left ventricular mass index; NT-proBNP, N Terminal-pro brain natriuretic peptide; PAF, paroxysmal atrial fibrillation; PCI, percutaneous coronary intervention; RAS, renin–angiotensin system; SPT, spasm provocation test; SR, sinus rhythm; UCG, ultrasound cardiography.

### 3.2 CAG, SPT, and CMFT Results between AF and SR Vessels

CMFTs were not measured in 60 vessels because of the small RCA (n = 7), inability to engage the catheter properly (n = 16), inability to insert the pressure wire into the targeted coronary artery (n = 5), the attending physicians’ decision (n = 29), and trouble with measuring equipment (n = 3). Hence, CMFT measurements were performed in 186 vessels. Table 2 summarizes the CAG, SPT, and CMFT results based on the vessels.

**Table 2.** **Results for CAG, SPT, and CMFT between AF and SR vessels**.

		SR-1 vessels	AF vessels	*p*-value
No. (%)		165 (89)	21 (11)	
LAD/RCA		105/65	11/10	0.316
Drugs provocative				
	ACh L/M/H	15/54/96	2/6/13	0.929
	Additional use of EM (%)	37 (22)	5 (24)	0.886
Atherosclerosis (%)		57 (35)	8 (38)	0.748
VSA (%)		90 (56)	10 (48)	0.455
Endotype of the coronary spasm				
	Focal/Diffuse/MVS/None/ND	40/50/20/48/7	9/1/6/5/0	0.021
	Presence of focal spasm (%)	40 (24)	9 (43)	0.068
	Presence of diffuse spasm (%)	50 (30)	1 (5)	0.014
Heart rate at CMFT (bpm)		72 ± 13	110 ± 22	<0.001
Pa during ATP infusion (mmHg)		93 ± 17	93 ± 15	0.853
(Numbers of vessels we were able to measure)		(140)	(14)	
Pd during ATP infusion (mmHg)		88 ± 18	88 ± 16	0.972
(Numbers of vessels we were able to measure)		(140)	(14)	
Tmn at baseline (seconds)		1.02 ± 0.48	0.78 ± 0.47	0.047
(Numbers of vessels we were able to measure)		(151)	(19)	
Tmn during ATP infusion (seconds)		0.37 ± 0.25	0.35 ± 0.11	0.770
(Numbers of vessels we were able to measure)		(151)	(19)	
Baseline Pd/Pa		0.98 ± 0.04	0.98 ± 0.04	0.619
FFR		0.94 ± 0.06	0.94 ± 0.06	0.977
CFR		3.2 ± 1.5	2.4 ± 1.5	0.035
	CFR of <2.0 (%)	32 (19)	11 (52)	0.001
IMR		30.7 ± 21.3	30.2 ± 11.1	0.918
	IMR ≥25 (%)	83 (50)	13 (62)	0.316
CMD (%)		92 (56)	14 (67)	0.342

Data are expressed as frequencies (percentages or numbers in some data). Abbreviations: ACh, acetylcholine; AF, atrial fibrillation; ATP, adenosine triphosphate; CAG, coronary angiography; CFR, coronary flow reserve; CMD, coronary microvascular dysfunction; ND, not diagnosed; CMFT, coronary microvascular function test; EM, ergonovine maleate; FFR, fractional flow reserve; IMR, index of microcirculatory resistance; LAD, left anterior descending coronary artery; L/M/H, low/moderate/high dose of acetylcholine; MVS, microvascular spasm; ND, not diagnosed; No., numbers; Pa, aortic pressure; Pd, distal pressure; RCA, right coronary artery; SPT, spasm provocation test; SR, sinus rhythm; Tmn, transit mean time; VSA, vasospastic angina.

**Fig. 4.**
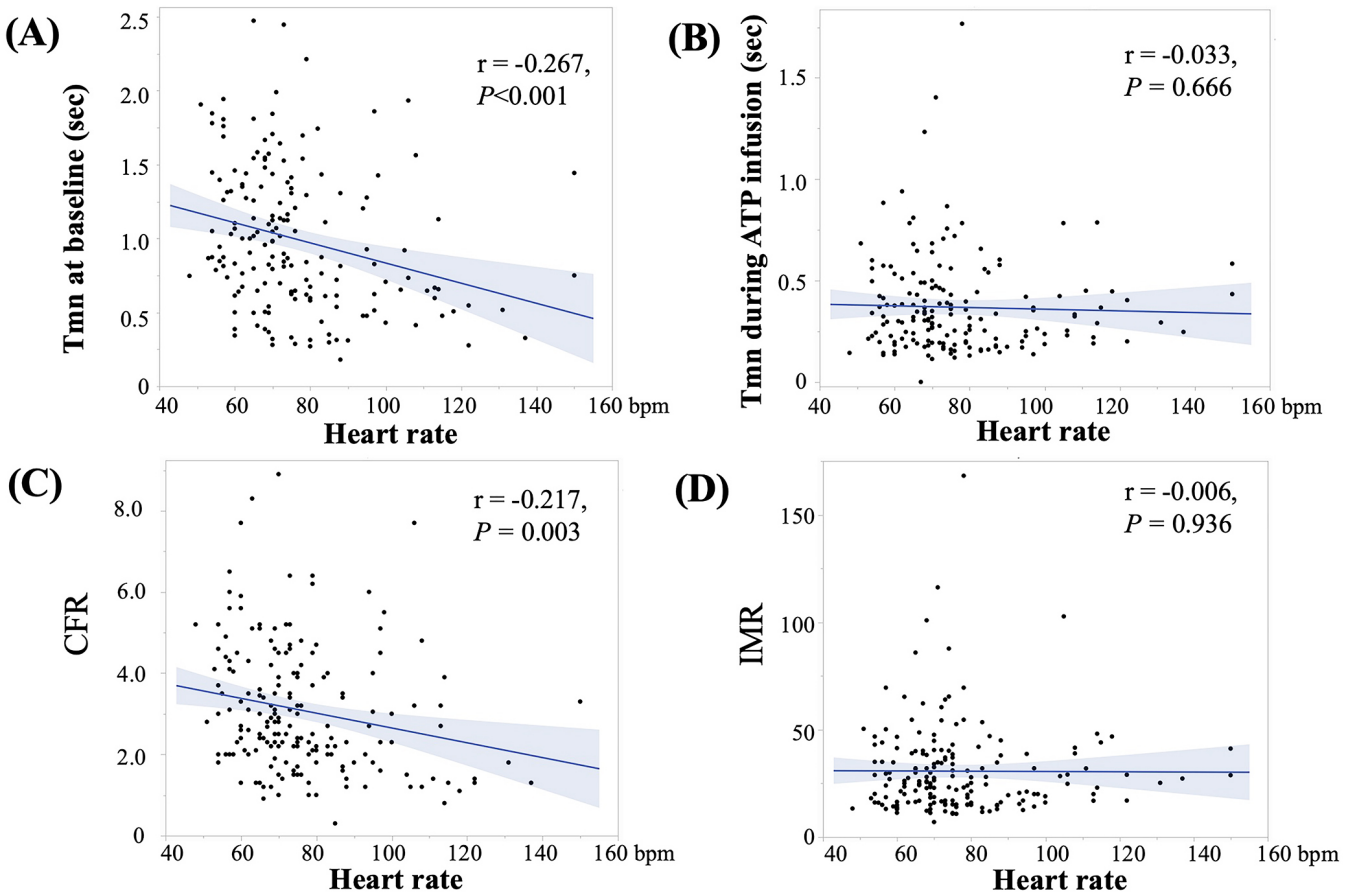
**Association between heart rate and Tmn at baseline (A), Tmn during ATP infusion (B), CFR (C), and IMR (D)**. Heart rate was negatively associated with Tmn at baseline (r = –0.267, *p* < 0.001) and CFR (r = –0.217, *p* = 0.003) but not with Tmn during ATP infusion (r = 0.033, *p* = 0.666) or IMR (r = –0.006, *p* = 0.936). Blue lines indicate confidence intervals. Abbreviations: ATP, adenosine triphosphate; CFR, coronary flow reserve; IMR, index of microcirculatory resistance; Tmn, transit mean time.

This study demonstrated 21 (11%) AF vessels and 165 (89%) SR-1 vessels. No significant differences were observed in LAD or RCA vessels, ACh dosage and additional EM administration, atherosclerosis presence, or coronary spasm frequency. Significant differences were observed between AF and SR-1 vessels in the coronary spasm endotypes (*p* = 0.021), with the incidence of diffuse spasm being lower in AF vessels (*p* = 0.014). The heart rate at the CMFT assessment was significantly higher in the AF vessels than in the SR-1 vessels (*p* < 0.001). The mean blood pressure in Pa and Pd during ATP infusions was comparable between the two groups. Tmn at baseline was significantly shorter in the AF vessels than in the SR-1 vessels (*p* = 0.047). However, Tmn during ATP administration was not different between the two groups. The baseline Pd/Pa, FFR, and IMR did not differ between the two groups. However, CFR was significantly lower in the AF vessels than in the SR-1 vessels (*p* = 0.035), and the frequency of CFR of <2.0 was higher in the AF vessels than in the SR-1 vessels (*p* = 0.001). The frequency of IMR when ≥25 and CMD did not differ between the two groups. Factors associated with CFR of <2.0 were AF rhythm (*p* < 0.001), endotype of coronary spasm (*p* = 0.036), and heart rate (*p* < 0.001). Logistic regression analysis revealed that heart rate was a significant factor for a CFR of <2.0 (*p* = 0.038, R^2^ = 0.100). Heart rate negatively correlated with Tmm at baseline (r = –0.267, *p* < 0.001) and with CFR (r = –0.217, *p* = 0.003) but not with Tmn during ATP infusion (r = –0.033, *p* = 0.666) or IMR (r = –0.006, *p* = 0.936, Fig. 4). The association between heart rate, Tmm at baseline, and CFR was separately investigated in SR-1 and AF vessels. Heart rate was negatively and significantly correlated with the Tmm at baseline (r = –0.221, *p* = 0.006) and the CFR (r = –0.162, *p* = 0.038) in the SR-1 vessels (n = 165), whereas no correlation was found between heart rate and the Tmm at baseline (r = –0.278, *p* = 0.254) or the CFR (r = –0.145, *p* = 0.529) in the AF vessels.

### 3.3 CAG, SPT, and CMFT Results between AF Vessels, AF-SR Vessels, and SR-2 Vessels

**Fig. 5.**
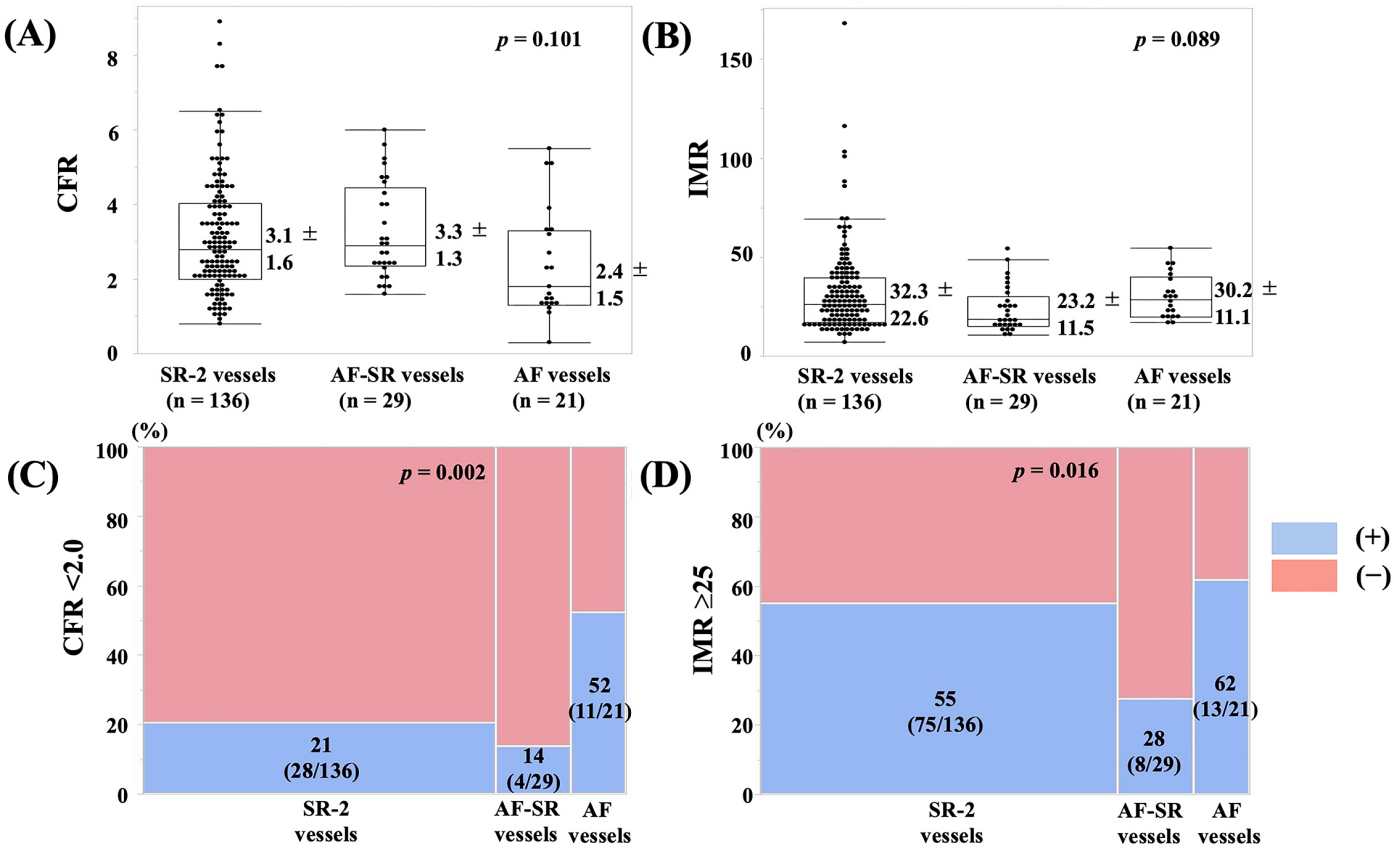
**The values of (A) CFR and (B) IMR and the frequencies of (C) CFR of <2.0 and (D) IMR of ≥25 in the SR-2 vessels, AF-SR vessels, and AF vessels**. CFR was not different in the three groups (*p* = 0.101), but the frequency of CFR of <2.0 was significantly higher in the AF vessels (*p* = 0.002). The IMR tended to differ in the three groups (*p* = 0.089), and the frequency of IMR of ≥25 was significantly lower in the AF-SR vessels (*p* = 0.016). Abbreviations: AF, atrial fibrillation; CFR, coronary flow reserve; IMR, index of microcirculatory resistance; SR, sinus rhythm.

Based on the subgroups of vessels, 21 (11%) were AF vessels, 29 (16%) were AF-SR vessels, and 136 (73%) were SR-2 vessels (Fig. 2 and Table 3). No significant differences were observed in LAD or RCA vessels, ACh dosage and additional EM administration, atherosclerosis presence, or coronary spasm frequency. A significant difference in the coronary spasm endotypes was observed among the three groups (*p* = 0.004). The heart rate at CMFT examination was significantly higher in the AF vessels group than in the other two groups (*p* < 0.001). The mean blood pressure in Pa and Pd during ATP infusions was comparable in the three groups. Tmn at baseline was significantly shorter in the AF and AF-SR vessels than in the SR-2 vessels (*p* = 0.012). Tmn during ATP infusion varied in the three groups (*p* = 0.028), and it was shorter in the AF-SR vessels than in the SR-2 vessels. Baseline Pd/Pa and FFR were comparable in the three groups. CFR was not significantly different in the three groups (*p* = 0.101, Fig. 5), but the frequency of CFR of <2.0 was significantly different in the three groups (*p* = 0.002). IMR tended to be lower in the AF-SR vessels than in the other two groups (*p* = 0.089). The frequency in IMR of ≥25 was significantly lower in the AF-SR vessels than in the other two groups (*p* = 0.016). Finally, CMD frequency tended to be lower in the AF-SR vessels than in the other two groups (*p* = 0.065).

**Table 3.** **Results for CAG, SPT, and CMFT between AF vessels, AF-SR vessels, and SR-2 vessels**.

		SR-2 vessels	AF-SR vessels	AF vessels	*p*-value
No. (%)		136 (73)	29 (16)	21 (11)	
LAD/RCA		87/49	18/11	11/10	0.594
Drugs provocative					
	ACh L/M/H	15/40/81	0/14/15	2/6/13	0.184
	Additional use of EM (%)	32 (24)	5 (17)	5 (24)	0.779
Atherosclerosis (%)		46 (34)	4 (38)	8 (38)	0.869
VSA (%)		75 (56)	15 (58)	10 (48)	0.746
Endotype					
	Focal/Diffuse/MVS/None/ND	31/44/16/42/3	9/6/4/6/4	9/1/6/5/0	0.004
	Presence of focal spasm (%)	31 (23)	9 (31)	9 (43)	0.125
	Presence of diffuse spasm (%)	44 (32)	6 (21)	1 (5)	0.021
Heart rate at CMFT (bpm)		71 ± 13	76 ± 13	110 ± 22	<0.001
Pa during ATP infusion (mmHg)		92 ± 18	94 ± 15	93 ± 15	0.901
(Numbers of vessels we were able to measure)		(116)	(24)	(14)	
Pd during ATP infusion (mmHg)		88 ± 18	88 ± 15	88 ± 16	0.998
(Numbers of vessels we were able to measure)		(116)	(24)	(14)	
Tmn at baseline (seconds)		1.06 ± 0.49	0.83 ± 0.41	0.78 ± 0.47	0.012
(Numbers of vessels we were able to measure)		(124)	(27)	(19)	
Tmn during ATP infusion (seconds)		0.39 ± 0.26	0.26 ± 0.12	0.35 ± 0.11	0.028
(Numbers of vessels we were able to measure)		(124)	(27)	(19)	
Baseline Pd/Pa		0.98 ± 0.04	0.98 ± 0.03	0.98 ± 0.04	0.844
FFR		0.94 ± 0.06	0.93 ± 0.07	0.94 ± 0.06	0.746
CFR		3.1 ± 1.6	3.3 ± 1.3	2.4 ± 1.5	0.101
	CFR of <2.0 (%)	28 (21)	4 (14)	11 (52)	0.002
IMR		32.3 ± 21.3	23.2 ± 11.5	30.2 ± 11.1	0.089
	IMR of ≥25 (%)	75 (55)	8 (28)	13 (62)	0.016
CMD (%)		81 (60)	11 (38)	14 (67)	0.065

Data are expressed as frequencies (percentages or numbers for some data). Abbreviations: ACh, acetylcholine; AF, atrial fibrillation; ATP, adenosine triphosphate; CAG, coronary angiography; CFR, coronary flow reserve; CMD, coronary microvascular dysfunction; CMFT, coronary microvascular function test; EM, ergonovine maleate; FFR, fractional flow reserve; IMR, index of microcirculatory resistance; LAD, left anterior descending coronary artery; L/M/H, low/moderate/high dose of acetylcholine; MVS, microvascular spasm; ND, not diagnosed; No., numbers; Pa, aortic pressure; Pd, distal pressure; RCA, right coronary artery; SPT, spasm provocation test; SR, sinus rhythm; Tmn, transit mean time; VSA, vasospastic angina.

## 4. Discussion

This retrospective observational study investigated the clinical characteristics of patients with ANOCA who developed AF when first treated with SPT with ACh, followed by CMFT treatment. Approximately 25% of the patients developed PAF, but our study could not determine any clinical characteristics that would predispose patients to PAF. Additionally, we examined the effect of AF on subsequent CMFT. Our results revealed that patients with persistent AF demonstrated little effect on IMR despite a reduced CFR caused by increased coronary blood flow velocity at rest. Conversely, CFR and IMR may be affected in patients who developed PAF but recovered to SR, indicating an improved coronary microcirculation. Moreover, caution should be exercised when interpreting SPT results using ACh preceded by CMFT because AF complications may affect CMFT measurements.

PAF occurred in approximately 8%–17% of the patients in the SPT using the ACh group [10, 11]. This was more predominant in RCA provocations [10, 11]. This could be caused by the differences in the distribution of muscarinic receptors in the coronary arteries [22]. The present study revealed PAF in 23% of the patients receiving RCA and in 6% administered with LCA, indicating that PAF was more prevalent in the RCA provocation. Our study observed provocation was more common than previously reported [10, 11], which may be caused by the definition of AF used and because we included patients with PAF of short durations (such as those that were brief and quickly restored to SR). Additionally, the definition of low, moderate, and high doses of ACh differed from study to study, and these definitions must be cautiously interpreted, but it did not occur at higher ACh doses as reported by Sueda *et al*. [23]. Few studies have examined which patients are more likely to develop PAF during SPT with ACh [11]. Saito *et al*. [11] revealed that PAF is more likely to occur in patients with a PAF history and low body mass index (BMI). The present study revealed that patients with PAF reported no PAF history, and BMI did not differ between patients with or without PAF. The differences in the groups studied may explain the differences in patients with ANOCA. Saito *et al*. [11] conducted a univariate analysis revealing that men were less likely to have PAF, but the differences were not significant in the multivariate analysis. The present study indicated no such significant differences but observed a trend toward a higher PAF incidence in women. Further research is warranted to understand the sex-related differences in PAF complications during the SPT in a multicenter registry. The present study revealed a trend toward lower NT-proBNP and echocardiographic E/e’ in the PAF group, although the differences were not statistically significant. These results indicated that patients with less severity of cardiac organic abnormalities may be more prone to PAF caused by ACh provocation. Additionally, MB also tended to be more prevalent in the PAF group. An association between MB and PAF during the operation was observed in patients with hypertrophic cardiomyopathy [24]. More research is required to clarify the associations of NT-proBNP, E/e’, and MB with PAF during ACh provocation. Additionally, no significant association was observed between the occurrence of AF and coronary spasms, based on previous reports and our research results [10, 11]. Our study revealed that diffuse spasm was less common in AF vessels than in SR-1 vessels among the endotypes of coronary spasm. The number of cases in a multicenter registry or other methods to investigate this issue needs to be increased because of the small number of cases of AF vessels.

Several reports [12, 13, 14, 15] on the effects of AF on coronary microcirculation have reported that increased heart rate with AF results in a faster coronary blood flow velocity at baseline, causing a lower CFR. The present study revealed no difference in the maximal coronary blood flow velocity under ATP infusion although the resting coronary blood flow velocity increased with increased heart rate. Additionally, no significant changes were observed in blood pressure at baseline (data not shown) and during ATP infusion. Thus, an increased heart rate was the only significant factor responsible for a reduced CFR on logistic regression analysis. Our data were consistent with the results of previous studies [12, 13, 14, 15]. However, the results were somewhat different when the correlations between heart rate and Tmm at baseline and CFR were investigated separately for SR-1 and AF vessels. Significant negative correlations between heart rate and Tmm at baseline and CFR were observed in SR-1 vessels, whereas no significant correlations between them were found in AF vessels. Factors other than simply increased heart rate, such as irregular rhythm, may be involved in the shortening of the Tmm at baseline and the decrease in CFR although the small number of AF vessels may contribute to this result. In any case, the number of cases of AF vessels is small and should be increased in the future. Myocardial ischemia has been reported in animal models of AF complicated by significant coronary artery stenosis [13]. However, we considered that no apparent myocardial ischemia was induced during AF because the patients in our study demonstrated no significant stenosis in the coronary arteries and no significant decreases in intracoronary pressure (Pd) or FFR during ATP infusion. Increased heart rate (although not AF) increases CFR variability, indicating that it could be a reliable IMR indicator in such cases [25]. The present study revealed that IMR was unaffected by the heart rate and did not significantly differ between the AF and SR vessels. Many cases revealed that an increase in IMR does not always coincide with a decrease in CFR in clinical practice [16, 26]. Therefore, IMR alone cannot diagnose CMD, but it is recommended to refer to the IMR value when the CFR decreases during the AF duration.

The coronary microcirculation in vessels that recovered quickly from PAF to SR was interesting. CFR was preserved in these vessels and IMR appeared lower, although this was not statistically significant. Consequently, a lower rate of having the two components of CMD was observed with CFR of <2.0 and IMR of ≥25. The vessels with preserved CMF can be quickly restored to SR after an initial AF. However, no differences in patient background between the three groups were observed, making it unlikely that CMF was preserved in such patients. Hence, a compensatory improvement in the coronary microcirculation during the stages of PAF and immediate recovery. Reportedly, coronary microcirculation does not restore immediately after electrical cardioversion in a clinical setting [15]. Additionally, whether CMF improves immediately after PAF is restored to SR is speculative, but a compensatory effect may be possible if the AF duration is extremely short. The number of such vessels was not large, the PAF duration was not measured and varied, and we cannot deduce how long the improvement in coronary microcirculation would last because this was a cross-sectional study. These issues could be resolved by increasing the number of patients in a multicenter registry or by investigating CMF in patients with AF-SR. In any case, coronary microcirculation should be carefully assessed even in vessels in which AF is restored to SR.

Clinically, this study implies that SPT with ACh first followed by CMFT was effective in improving the rate of coronary spasm identification, but concomitant AF may affect the results of subsequent CMFT. IMR values are advisable to be referred for CMD diagnosis because CFR decreases with increased heart rate in cases of persistent AF. Diagnosing CMD may be difficult if PAF is restored to SR immediately after it occurs. In any case, the CMFT should be interpreted with caution when AF occurs. AF frequently occurs during ACh provocation of the RCA. Performing SPTs and CMFTs in the LCA and RCA may increase the diagnostic yield of VSA and CMD [27], but limiting them to the LCA could be a countermeasure [26].

This study has some limitations. First, this was a single-center, retrospective study and the number of patients with persistent AF or with AF restoring to SR was quite small. Additionally, many cases underwent CMFT in only one coronary artery at the attending physician’s discretion. The application of these results is unclear internationally. Second, recording during CMFT measurements with intracoronary pressure and Tmn data missing in some cases was insufficient. Third, none of the patients in this study reported a PAF history, as far as we could identify from their medical records and interviews. However, the method of confirmation may not be sufficient because Holter ECGs were not performed frequently enough. Fourth, LAD and RCA were analyzed together because the number of AF cases was not large in this analysis. However, RCAs demonstrated generally higher Tmn and higher IMR than LADs [28]. Our study revealed no differences in the frequency of LAD and RCA measurements between the AF and SR groups, which did not influence the present results, but analyzing LAD and RCA separately for each vessel as they should seem correct. This is another issue that should be considered with an increased number of cases in a multicenter registry or other studies. Finally, the data were obtained from a cross-sectional study, and the CMFT data evolution in patients with AF was not observed. A method is used to measure CMFT after electrical cardioversion or administration of antiarrhythmic drugs by restoring AF to SR, but restoration of AF to SR is only performed after completing the examination due to the effects of these procedures and drugs on coronary microcirculation and because of time constraints.

## 5. Conclusions

We focused on AF which occurs when SPT with ACh is utilized for chest pain screening in patients with ANOCA in the present study. PAF occurred in 25% of the patients in our protocol. Our study did not reveal any particular clinical features in the background of patients that would predispose these patients to PAF development. We then examined whether AF would affect the results of the subsequent CMFT and the results indicated that CFR decreased with increasing heart rate in cases of persistent AF, but IMR was not related to heart rate. CMD with reference to IMR is recommended to be evaluated when AF is persistent and CFR declines. Conversely, CFR was preserved and IMR was low in vessels where AF was quickly restored to SR. This indicates retained coronary microcirculation. Subsequent interpretation of CMFTs should be done cautiously when AF occurs during SPT with ACh, whether persistent or not. The results of this study warrant validation in a multicenter registry.

## Data Availability

Data and materials inquiries can be directed to the corresponding author.

## Artificial Intelligence (AI) Policy

No AI was used in the preparation of this paper.

## Abbreviations

ACh, acetylcholine; AF, atrial fibrillation; ANOCA, angina with non-obstructive coronary artery disease; ATP, adenosine triphosphate; CAD, coronary artery disease; CAG, coronary angiography; CFR, coronary flow reserve; CKD, chronic kidney disease; CMD, coronary microvascular dysfunction; CMF, coronary microvascular function; CMFT, coronary microvascular function test; DM, diabetes mellitus; eGFR, estimated glomerular filtration ratio; EM, methylergometrine maleate; FFR, fractional flow reserve; H, high dose; IMR, index of microcirculatory resistance; L, low dose; LAD, left anterior descending coronary artery; LCA, left coronary artery; LVEF, left ventricular ejection fraction; LVMI, left ventricular mass index; M, moderate dose; MB, myocardial bridging; MVS, microvascular spasm; n, number; ND, not diagnosed; NTG, nitroglycerin; No., number; NT-proBNP, N Terminal-pro brain natriuretic peptide; Pa, aortic pressure; PAF, paroxysmal atrial fibrillation; PCI, percutaneous coronary intervention; Pd, distal pressure; RAS, renin–angiotensin system, RCA, right coronary artery; SPT, spasm provocation test; SR, sinus rhythm; Tmn, transit mean time; UCG, ultrasound cardiography; VSA, vasospastic angina.
